# Streamlining wearable data integration for EHDS: a case study on advancing healthcare interoperability using Garmin devices and FHIR

**DOI:** 10.3389/fdgth.2025.1636775

**Published:** 2025-10-06

**Authors:** Somayeh Abedian, Eugene Yesakov, Stanislav Ostrovskiy, Rada Hussein

**Affiliations:** ^1^ Ludwig Boltzmann Institute for Digital Health and Prevention, Salzburg, Austria; ^2^ Institute of Health Policy, Management and Evaluation, Dalla Lana School of Public Health, University of Toronto, Toronto, ON, Canada; ^3^ Edenlab, Innovative Digital Health Solutions, Tallinn, Estonia

**Keywords:** Patient-Generated Health Data (PGHD), FHIR (Fast Healthcare Interoperability Resources), healthcare interoperability, wearable devices, data integration

## Abstract

**Introduction:**

Patient-Generated Health Data (PGHD) collected through wearable devices such as smartwatches offers new opportunities for personalized care, chronic disease management, and preventive health. Despite this potential, technical, regulatory, and interoperability challenges still limit the integration of PGHD into healthcare systems, especially in relation to standards such as Fast Healthcare Interoperability Resources (FHIR) and the European Health Data Space (EHDS).

**Methods:**

This study used the Garmin Vívoactive 4 smartwatch to collect PGHD and integrate it into a FHIR server via the Fitrockr hub and API. The Kodjin FHIR server was deployed to enable standardized data storage and transfer. In parallel, data from the Modular Open Research Platform (MORE) were examined for compatibility with FHIR resources. The process included device enrolment, data collection, mapping to FHIR specifications, and evaluation of compliance with General Data Protection Regulation (GDPR) requirements.

**Results:**

The prototype demonstrated that data from Garmin devices could be securely collected, mapped, and transferred into a FHIR environment. Integration through the Fitrockr hub ensured structured data formatting and reliability. The analysis of PGHD from the MORE platform confirmed that heterogeneous data types, including physiological measures and survey responses, could be represented with appropriate FHIR resources. These findings highlight the technical feasibility and scalability of PGHD integration.

**Discussion:**

The results confirm that PGHD from wearable devices can be standardized and transferred into healthcare systems in compliance with international standards and European regulations. This approach contributes to bridging the gap between personal health data and medical decision-making, supporting the objectives of the EHDS and enabling further use of PGHD in research and innovation.

## 1 Introduction

The increasing frequency of wearable health devices grants new chances and opportunities for integrating Patient-Generated Health Data (PGHD) into clinical workflows. Wearable technologies, such as Garmin smartwatches, generate continuous health metrics that can provide valuable insights for personalized healthcare, continuing disease management, and preventive care (1–5). Many research studies and systematic reviews display the effect of these types of data in promoting wellness and helping disease management in the clinical and health domains (6–8).

Despite PGHD's potential, several technical, regulatory, and usability barriers hinder effective PGHD utilization in clinical or medical decision making (9).

However, the researchers show some risks and challenges related to legal validation, privacy, or data sharing, including counting this type of data in medical records as clinical data, and in addition, some barriers in interoperability between wearable devices and standards in the healthcare domain, such as Fast Healthcare Interoperability Resources (FHIR®) (10–13). The FHIR standard has been recognized as a widely accepted standard to facilitate structured health data exchange across different systems, supporting the integration of PGHD into Electronic Health Records (EHRs) (14).

On the other hand, PGHD is vital to the European Health Data Space (EHDS), as it provides a more complete view of patient health. Integrating data from devices and apps enables personalized care and enhances research, ultimately improving healthcare outcomes. Thus, the EHDS regulation highlights the need for robust interoperability frameworks to allow the secure sharing and exchange of health data across countries (15).

Furthermore, the Digital Health Convener (DH-Convener) initiative, conceptualized by the Ludwig Boltzmann Institute for Digital Health and Prevention, aims to create interoperability and security as a service platform that integrates PGHD with EHRs (16, 17) in alignment with the EHDS technical and governance requirements for the secondary use of data. This study addresses a prototyping of the DH-Convener initiative for PGHD interoperability. We selected one popular model of Garmin, Vívoactive 4. It is widely used in healthcare research based on its ability and other potential to provide consistent real-time data as a reliable process on various health metrics (18).

Garmin Vívoactive 4 is also considered a practical alternative to gold-standard ECG procedures for heart rate variability measurements, with most markers in the same range (19).

These results support the consistency of Garmin Vívoactive 4 and its reliability in capturing accurate health data, making it a valuable tool in healthcare research as well.

Thus, the DH-Convener prototype provides a PGHD integration model using Garmin Vívoactive 4 smartwatches, focusing on real-time data streams from a PGHD hub (collected by the Fitrockr platform) and based on the FHIR standard (stored by the Edenlab FHIR server) in compliance with GDPR.

As Patient-Reported Outcomes and questionnaires are considered valuable PGHD resources, we also integrated the Ecological Momentary Assessment (EMA) collected in research studies using the Modular Open Research Platform (MORE). MORE is a research infrastructure designed to support digital health studies. It consists of a web-based study management application, which allows researchers to configure studies and specify the data to be collected, and a companion study app that helps guide and support participants throughout long-term studies. MORE facilitates data collection in real-world settings, enabling researchers to analyze PGHD, and it actually works as a PGHD data hub (20, 21).

**Figure 1:**
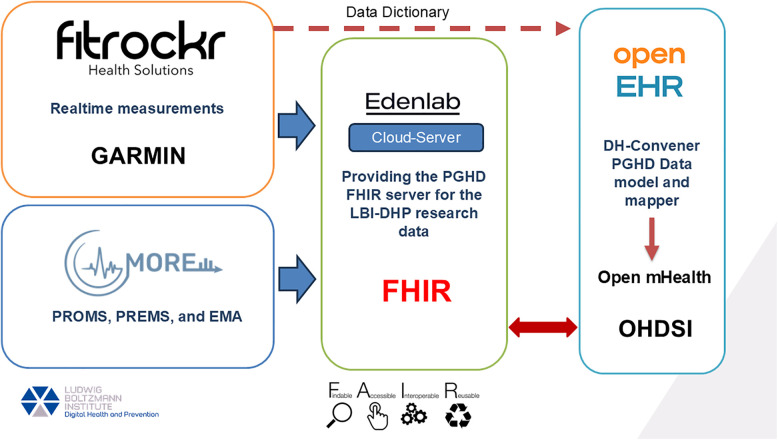
Integrating wearable data with DH-convener and PGHD solutions in healthcare interoperability standards. “Fitrockr” logo reproduced with permission from https://www.fitrockr.com/, “MORE” app logo reproduced with permission from https://dhp.lbg.ac.at/more/?lang=en, “Edenlab” logo reproduced with permission from https://edenlab.io/ and “Open EHR” logo reproduced with permission from https://openehr.org/.

We analyzed the collected data and mapped them to the FHIR standard (see Figure 1).

This paper summarizes the results of our prototyping in this endeavor, utilizing FHIR in standardizing the PGHD in alignment with the EHDS recommendations for the secondary use of data in research and innovation (22).

The prototyping process included several key steps: collecting data, configuring the FHIR server, operating the Fitrockr API, technical mapping PGHD from the Garmin device to FHIR specifications, and developing the portal. Moreover, the evaluation and analysis of data elements from the MORE platform are needed for conformance compatibility with FHIR resources. Ultimately, the aim was to demonstrate a scalable method for incorporating wearable health data from various sources into healthcare systems.

## 2 Methods and materials

**Figure 2:**
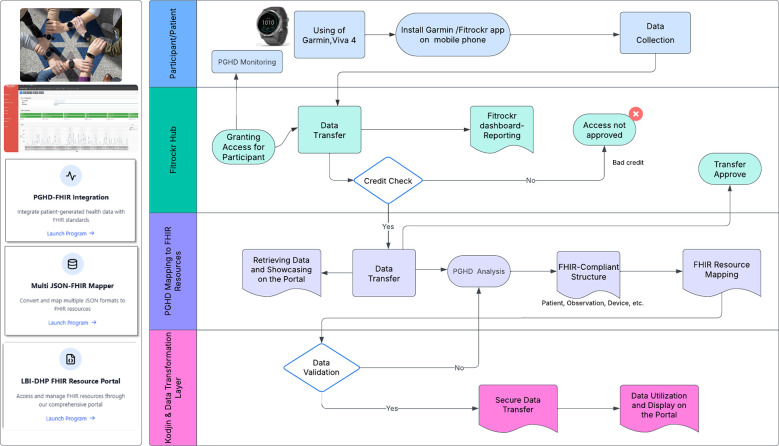
Integrating PGHD from Garmin into FHIR through data transformation layers.

This study follows a multi-step method to integrate wearable health data into the FHIR ecosystem as a case study. The integration process involves several key workings: data collected from Garmin Vívoactive 4 smartwatches via the Fitrockr API, data transformation into FHIR-compliant resources, and secure data transfer to an FHIR repository. Figure 2 illustrates overall workflow.

Alongside this, because MORE is a platform for secondary use and research of health data, we attempted to assess the compatibility of PGHD, which is integrated into the platform, using the FIHR standard, as an experimental study on the secondary use of data platform on health data.

Consequently, we received a JSON output of the MORE platform and based on FHIR resources and the FHIR Implementation Guide (IG), we provided an analysis of the FHIR-compliant schema (23). Details of these processes are in the following.

### 2.1 Seamless integration: leveraging PGHD from Garmin device for FHIR interoperability

This section outlines the process of integrating PGHD from the Garmin Vívoactive 4 into an FHIR server (similarly for transferring data to an FHIR-compatible health system), preparing the data for transfer to other healthcare systems or EHRs. It includes connecting devices, collecting data, mapping PGHD to FHIR standards, and deploying the FHIR server to ensure seamless interoperability and maintain data integrity, as detailed in the following sections:.

#### 2.1.1 Device enrollment and data collection

In this phase, we selected Garmin Vívoactive 4 smartwatches for their ability to track key PGHD, including heart rate, step count, sleep patterns, activity intensity, stress level, etc.

Once the Garmin Vívoactive 4 devices were enrolled, they were set up to continuously track and record health metrics which are vital for monitoring an individual's physical activity, sleep quality, burning calories, body composition, and overall health. This setup involved adjusting the data sampling rates and ensuring the devices could automatically sync with the central system through the Garmin Connect tool, which ensured a smooth and nonstop flow of data.

On the other hand, real-time monitoring was essential for tracking important health measures. Therefore, the Garmin Connect tool was used to configure the devices, making sure they were correctly set up to gather data while also ensuring that the data synced accurately with the central system for reliable and constant data transfer.

#### 2.1.2 Integrating PGHD from garmin vívoactive 4 with FHIR compatibility

Aligned to better management of data collection, the Fitrockr hub was employed. The Fitrockr hub served as the central platform for data acquisition, where it securely gathered and organized the data from the Garmin devices. This platform has been shown to enhance data integration by supporting various wearable technologies and ensuring secure data administration (24).

The collected data was then prepared for integration with the FHIR server. A critical component of this process was using the Fitrockr data dictionary, which provided a list of standardized data elements for the PGHD from wearable tools (25). In other words, it provides a clear point of view on wearable health data, and then defining details of interoperability between different systems will be easier.

The Fitrockr data dictionary provides structured naming and codification of PGHD variables derived from Garmin and other wearable devices. The codification primarily relies on vendor-specific identifiers, using parameter names and structures defined in the Garmin Health API (25), which are then systematically organized within Fitrockr's internal data dictionary (26). This enables structured extraction of key health metrics such as heart rate, step count, and sleep stages. However, the data dictionary does not currently use clinical coding systems such as LOINC or SNOMED CT, limiting direct clinical interoperability.

The data dictionary is maintained and validated internally by the Fitrockr team, who test its accuracy and reliability through various production-level integrations. While not externally certified, the data schema is stable and widely used in Fitrockr's deployments for research and wellness analytics.

Although it is not officially integrated with the HealthData@EU framework, the Fitrockr dictionary, due to its structured formatting and FHIR mapping offers a model that could be adapted to align with the interoperability goals defined by the TEHDAS recommendations (22), especially in the context of secondary data use and PGHD harmonization within EHDS.

By utilizing the Fitrockr hub and its data dictionary, we ensured that all PGHD was accurately formatted, stored, and prepared for smooth integration into the healthcare ecosystem.

The hub also played a vital role in maintaining the integrity and quality of the data throughout the intake process, ensuring that it met the necessary standards for further analysis. If any errors occurred in the data, such as inconsistencies in format or unusual values, the hub would identify and correct these issues by using preprocessing and correction methods, such as fixing incorrect values or aligning the data with the correct formats.

**Figure 3:**
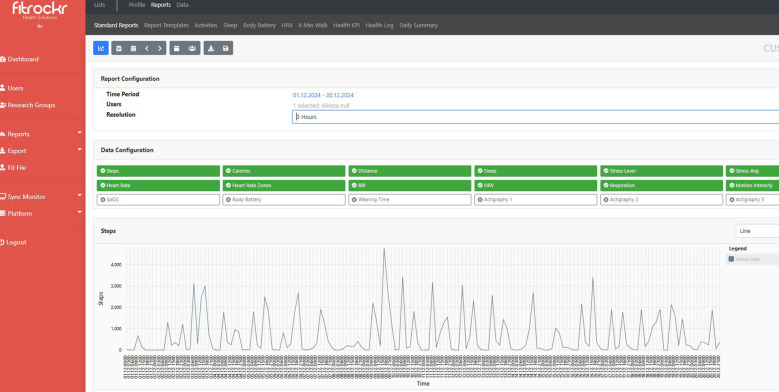
Overview of the Fitrockr dashboard for data monitoring and transfer trends.

This process ensured that the data remained accurate and ready for subsequent analysis, which in the next step involved mapping to FHIR standard resources. Additionally, users could monitor and view their data through the Fitrockr dashboard, which demonstrated the integrated data on the hub. Figure 3 illustrates a snapshot of the Fitrockr hub.

#### 2.1.3 FHIR server deployment and API configuration

The Kodjin FHIR server was selected to ensure standardized data storage and retrieval. It was configured to support seamless processing of FHIR resources and enable secure management of our prototyping on PGHD. The Kodjin solution uses a low-code declarative approach, allowing for efficient integration with minimal coding. We used a cloud version of the Kodjin FHIR server in this study.

To make the data ready for integration with the FHIR server, we used the secure Public Fitrockr API to access the collected PGHD from the Fitrockr hub and then mapped each data element individually and created an FHIR bundle, ensuring it was practical with the FHIR standard.

Moreover, the Public Fitrockr API was used for its secure, standardized access to Garmin data. Participants voluntarily initiated data sharing via the study portal. Token-based authentication and encrypted communication ensured GDPR-compliant, ethical use of PGHD in the research.

#### 2.1.4 PGHD mapping to FHIR resources

In this step, we mapped the processed and collected data to FHIR profiles using the Edenlab transformation tool to ensure seamless integration. In this process, key FHIR resource types were used, including Patient for personal information like name, gender, birth date, and contact details, and Observation for capturing health-related data such as Heart Rate, stress data, respiration, steps, VO2 max, Pulse Ox, BBI data, etc.

These resources were structured and linked appropriately, allowing consistent and standardized data exchange, aligning with the FHIR framework for smooth interoperability and integration into healthcare ecosystems.

We utilized the Kodjin mapping tool to convert the Garmin Vívoactive 4 PGHD collected on the Fitrockr hub into appropriate FHIR resources as a Liquid.

In fact, Liquid is a template language designed to work with FHIR resources data mapping. It enables complex mappings and offers flexibility for data processing tools. This facilitated unified mapping and ensured compatibility with the FHIR standard for further integration into healthcare systems (27).

**Figure 4:**
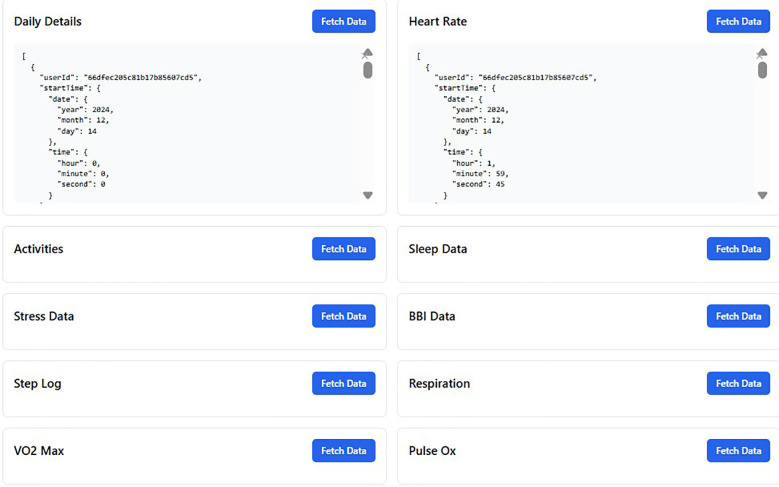
Retrieving data from the Fitrockr hub and mapping it to FHIR resources.

Some of the data we retrieved from the Fitrockr hub and mapped to FHIR resources is illustrated in Figure 4. In addition, to further demonstrate the data transformation process, we have included a raw JSON sample from the MORE research platform and its mapped version in FHIR format in the Supplementary Material.

#### 2.1.5 Validation and compliance monitoring

In this prototype, we developed a portal to allow users to control their data-sharing preferences. This web-based portal provides an option for them to exclude sensitive data, such as demographic details or stress analysis, and send only selected data to the server of their choice.

In this research-focused project, formal consent forms were not applied, as the study did not involve clinical intervention or treatment. Instead, we developed a participant-controlled portal that allowed individuals to fully access and review their wearable data collected through Fitrockr.

Data sharing only occurred when participants actively clicked a “Send to FHIR Server” button, indicating voluntary and informed participation in the research. This design supports the principle of active informed consent through action, ensuring transparency and autonomy.

To ensure privacy and compliance with the GDPR, pseudonymization was implemented during data transmission to the FHIR server, each participant was assigned a unique, non-identifiable code that replaced direct identifiers in the server. In the second part of the study, where we analyzed data exported from the MORE platform, no direct transmission occurred. These datasets were already anonymized due to their research nature, and no identifying information was included. As such, GDPR compliance was ensured through anonymization at source.

While this solution is specific to the research study, adopting GDPR-compliant practices is critical for larger-scale projects to protect user privacy and ensure data security by consulting with GDPR experts to ensure that the solutions meet the European regulation standards for data sharing, such as using short message (SMS) on participants mobile phones to give their confirmation before using related data.

At the same time, for verification and confirmation on the successful transfer of data to the FHIR server, we developed a quick dashboard that allows for retrieving and fetching PGHD based on FHIR identifiers such as Patient ID and Participant ID or Patient name, etc. Therefore, it serves as a tool and proof for verification of the entire process in our work.

### 2.2 Evaluation of compatibility of research data from the MORE platform with FHIR

We examined a subset of PGHD from the MORE research platform, which houses data from wearables like Polar; In the second section of our study, we worked with extracted JSON data to assess its conformance with the FHIR standard rather than utilizing an API for direct data transmission (as we did in the first section of the research when we employed an API to obtain data from the Fitrockr hub). This compatibility assessment was used in two stages:.

#### 2.2.1 Data extraction and preprocessing

We worked with a subset of JSON-formatted PGHD instead of utilizing an API. The extracted data was reviewed to determine the necessary transformations for mapping to FHIR resources.

In our analysis of the MORE research platform, we identified that the research data is categorized into four key elements:

• Heart Rate: Physiological data obtained from wearable devices.
• Acceleration: Motion data used for activity tracking like walking, running, or sedentary behavior.
• Survey Responses: User-reported questions and answers.
• GPS Data: Location information linked to recorded activities.

#### 2.2.2 Mapping to FHIR resources

To align the data with FHIR standards, we mapped each data type to the corresponding FHIR resources:

• Heart rate, GPS data, and acceleration → FHIR Observation
• Survey responses → FHIR QuestionnaireResponse

This transformation was implemented using the Kodjin Liquid templates to structure the extracted JSON data into FHIR-compliant formats.

## 3 Results

The integration of Garmin Vívoactive 4 smartwatches with an FHIR-compliant system through the Fitrockr hub has demonstrated significant technical feasibility for real-time data acquisition, transformation, and interoperability with healthcare systems. This section presents the key outcomes from the prototyping process.

### 3.1 Data collection and synchronization

The Garmin Vívoactive 4 devices successfully collected continuous personal health data, including heart rate, step count, sleep patterns, activity levels, and stress data. The devices were enrolled and configured to synchronize with the central system using the Garmin Connect tool, ensuring reliable data transfer. Data synchronization was achieved with minimal latency, allowing real-time tracking and continuous monitoring of the patient's health metrics. The devices provided highly consistent data with minimal errors, validating their use in clinical and research settings.

### 3.2 Fitrockr hub data integration

The Fitrockr hub played a pivotal role in the secure aggregation and preprocessing of data from the Garmin devices. Data quality was maintained by leveraging the Fitrockr data dictionary, which ensured consistency and compatibility between the various wearable technologies used in the study. Any inconsistencies in the data, such as missing or outlier values, were automatically flagged and corrected, maintaining data integrity for further analysis. This platform facilitated seamless data ingestion and ensured compliance with healthcare data security protocols.

### 3.3 Mapping data to FHIR resources

Data transformation from Garmin PGHD into FHIR-compliant resources was successfully completed using the Kodjin FHIR server and resource mapper. The key FHIR resources used for this process included Patient, Observation, and Bundle. PGHD elements such as heart rate, steps, sleep quality, and stress levels were mapped to FHIR Observation resources, while personal details were assigned to Patient resources. This ensured that data from various wearable devices, including Garmin, could be easily integrated into any FHIR-compliant healthcare system, ensuring interoperability with existing EHR systems.

### 3.4 Data compliance and GDPR considerations

A crucial aspect of the prototyping phase was ensuring that the collected PGHD adhered to data protection regulations, specifically GDPR. A user-friendly portal was developed to allow participants to control their data-sharing preferences, with the ability to decline some items of sharing, such as sensitive data like stress levels or personal demographics. This ensured user autonomy while maintaining compliance with European data privacy laws. Furthermore, consultations with GDPR experts confirmed that the solution met the regulatory standards required for large-scale health data integration projects.

### 3.5 Evaluation of compatibility with MORE platform data

The analysis of PGHD data from the MORE research platform demonstrated that even non-API data could be mapped to FHIR standards. We successfully transformed a subset of JSON-formatted data, including heart rate, acceleration, survey responses, and GPS data, into FHIR-compatible formats using Kodjin Liquid templates.

This demonstrated the versatility of the FHIR standard in accommodating data from various sources, including wearables not initially supported by FHIR-compliant APIs. The mapped data was categorized appropriately into Observation (for physiological and physical activity data), Questionnaire Response (for survey data), and other relevant FHIR resources.

### 3.6 Scalability and future integration

The scalability of the proposed solution was validated through the integration of PGHD from various wearable devices into the FHIR ecosystem. The use of the Fitrockr hub, Kodjin server, and FHIR resources demonstrated that this method could be applied to other wearable technologies, ensuring that PGHD from different sources can be standardized and integrated into healthcare systems seamlessly. This scalability is critical for the extensive approval of wearable health data in clinical and research settings.

## 4 Discussion

### 4.1 Aspects of interoperability in the DH-convener prototyping

In this prototype, we used of Fitrockr solution to collect PGHD streams from the Garmin Vívoactive 4. This ensures that the data is disciplined in a standardized format and machine readable to be transferred securely. To ensure consistency across various FHIR-compliant systems and platforms, we align the Fitrockr data dictionary with FHIR resources like Observation, Device, and Patient, PGHD that meet a suitable conversion into a standardized format, and so they are interpreted across various FHIR-compliant systems and platforms.

In the data exchange and integration concept, as shown in the picture, we examine how data flows fluently between wearable devices, the FHIR cloud server, and structured health data repositories. The FHIR standard serves as the link between these data sources, enabling smooth interoperability. This facilitates live access to PGHD within clinical or medical workflows, allowing healthcare providers to include wearable data in patient records and make informed medical decisions.

Additionally, alongside the Fitrockr hub, we employed the MORE research platform for the secondary use of PGHD, including Patient-Reported Outcome Measures (PROMs), Patient-Reported Experience Measures (PREMs), questionnaires, and other personal health data that can be defined by researchers and considered PGHD. We evaluated the compatibility of these data types with FHIR resources and mapped them to ensure compliance with FHIR standards. In this case, we assess the compatibility of the MORE platform's JSON data set with FHIR standards.

As a cross-system interoperability, in addition to FHIR, DH-Convener explores complementary standards like openEHR and OMOP for semantic interoperability and data representation. While FHIR ensures the exchange of data between systems, openEHR's archetype-based modeling provides a framework for representing complex clinical and health data in a structured and meaningful way.

The DH-Convener initiative, by providing PGHD in FHIR, openEHR, and OMOP standards, aims to ensure that PGHD is not only exchanged but also accurately represented and interpreted across various health information systems (16).

### 4.2 EHDS and PGHD interoperability

With the EHDS's entry into force on 26 March 2025 (28). PGHD plays a crucial role in fulfilling the EHDS goals of empowering individuals and enriching healthcare insights. With PGHD interoperability, the EHDS can gain a more comprehensive understanding of a patient's health status beyond traditional clinical settings.

This inclusion facilitates personalized care, promotes preventative measures, and enhances research capabilities, ultimately leading to improved patient outcomes and a more holistic approach to healthcare delivery.

The DH-Convener aligns with these objectives by demonstrating how FHIR-standardized PGHD can be integrated into EHR. The lessons learned from this integration and PGHD interoperability can inform the implementation of the EHDS national Health Data Access Bodies (HDABs) to efficiently manage and process diverse data sources while maintaining quality and consistency. Ultimately, the HDABs will regulate the secondary use of PGHD for research and innovation, ensuring privacy through strict rules involving pseudonymization and anonymization. Access requires HDAB permits, guaranteeing data is used ethically and legitimately.

Furthermore, Fitrockr's structured data acquisition and mapping practices provide valuable input for the development of future PGHD integration pipelines. In alignment with TEHDAS recommendations (22), such platforms demonstrate practical approaches for harmonizing and reusing non-clinical PGHD within cross-border health data infrastructures governed by the EHDS.

### 4.3 Future works and impact on healthcare systems

According to this study and operational work within the DH-Convener initiative, the following technical directions for future research and development are proposed:.

#### 4.3.1 Real-time data processing and AI-powered clinical decision support

The integration of real-time data processing frameworks for PGHD, especially in the context of PREMs and PROMs, should be prioritized for future efforts. This involves leveraging machine learning (ML) and artificial intelligence (AI) for live analysis of PGHD, enabling actionable insights and supporting Clinical Decision Support Systems (CDSS) (29–31). These advancements would enhance personalized care and precision medicine by utilizing live data from wearable devices to optimize care decisions. Expanding AI-driven predictive analytics for clinical outcomes will improve decision-making and treatment pathways, offering more targeted and effective healthcare interventions (32).

#### 4.3.2 Data security, privacy, and regulatory compliance for PGHD

Ensuring the security and privacy of sensitive health data is crucial in the integration of PGHD into the healthcare system. Since wearable devices are not classified as medical devices, the legal status and standards of PGHD are still under development, requiring further research on their quality, authentication, and authorization processes. The EHDS aims to establish a harmonized framework for health data sharing while ensuring strong data protection measures, particularly in compliance with the GDPR and other European laws (33).

Additionally, future work should emphasize developing robust encryption and privacy-preserving technologies, such as federated learning and especially homomorphic encryption, to protect PGHD during transmission and storage. Furthermore, systems for managing dynamic consent in compliance with EHDS and GDPR should be designed to ensure data security without compromising clinical utility (34). In this context, emerging technologies such as blockchain offer promising models for secure and transparent data sharing while maintaining patient consent and traceability. Recent researchs are exploring the disruptive potential of blockchain in managing health data compliance, particularly under GDPR (35).

## 5 Conclusion

The integration of PGHD using FHIR standards marks a significant step toward advancing patient-centered, data-driven healthcare. Through the DH-Convener initiative, the importance of interoperability, data quality, regulatory compliance, and clinical applicability in utilizing wearable data for improved health outcomes is underscored. As identified in this study, addressing the existing challenges by improving standardization, strengthening regulatory frameworks, and leveraging AI-driven analytics will be essential to unlocking the full potential of PGHD in the future of digital health.

The study applies the FAIR (Findable, Accessible, Interoperable, and Reusable) principles to ensure that PGHD collected from wearable devices are structured, standardized, and optimized for secondary use. To achieve findability, we aligned the data with well-defined metadata and standardized formats, allowing efficient identification and retrieval. Accessibility was ensured by securely storing the data while maintaining controlled and authorized access for clinical and research purposes. Mapping PGHD to FHIR resources significantly enhanced interoperability, allowing seamless data exchange between diverse healthcare systems and platforms.

Additionally, integrating the MORE research platform supported the reusability of PGHD for secondary analysis, aiding applications such as patient-reported outcomes (PROMs) and experience measures (PREMs).

## Data Availability

The raw data supporting the conclusions of this article will be made available by the authors, without undue reservation.

## Supplementary material

Supplementary Material 1 FHIR transaction bundle generated from MORE platform sample.
