# Supplementary material for: Streamlining wearable data integration for EHDS: a case study on advancing healthcare interoperability using Garmin devices and FHIR
Source: Front Digit Health. 2025 Oct 6;7:1636775. doi: 10.3389/fdgth.2025.1636775 (PMC12536430; doi:10.3389/fdgth.2025.1636775)
Supplement: Supplementary Material 1 — FHIR transaction bundle generated from MORE platform sample. [file Datasheet1.pdf]

Supplementary Material

1 MORE Research Platform JSON Snapshot Prior to FHIR Standard Mapping


```json
[
  {
    "firstName": "****",
    "lastName": "****",
    "age": 30,
    "email": "****"
  },
  {
    "observation": "blood_pressure",
    "value": 120,
    "unit": "mmHg",
    "date": "2024-03-14"
  },
  {
    "datapoint_id": "672c9bc8ecf4565f5cb19fd9",
    "participant_id": "participant_10",
    "study_id": "study_34",
    "study_group_id": "study_group_2",
    "observation_id": "2",
    "observation_type": "polar-verity-observation",
    "data_type": "polar-verity-observation",
    "storage_date": "2024-11-07T10:52:03.152316270Z",
    "effective_time_frame": "2024-11-07T10:51:52.887270Z",
    "data_hr": 103
  }
]
```


2 Final FHIR Bundle Based on JSON Snapshot of MORE Platform Research Data


```json
{
  "resourceType": "Bundle",
  "type": "transaction",
  "entry": [
    {
      "resource": {
        "resourceType": "Patient",
        "id": "d90iojp5v",
        "identifier": [
          {
            "system": "https://api.fitrockr.com",
            "value": "d90iojp5v"
          }
        ],
        "name": [
          {
            "family": "*****",
            "given": [
              "****"
            ]
          }
```

```
        ],
        "birthDate": "1995",
        "telecom": [
          {
            "system": "email",
            "value": "*****"
          }
        ]
      },
      "request": {
        "method": "PUT",
        "url": "Patient/d90iojp5v"
      }
    },
    {
      "resource": {
        "resourceType": "Observation",
        "id": "1ub982n82",
        "status": "final",
        "category": [
          {
            "coding": [
              {
                "system": "http://terminology.hl7.org/CodeSystem/observation-
category",
                "code": "vital-signs"
              }
            ]
          }
        ],
        "code": {
          "coding": [
            {
              "system": "http://loinc.org",
              "code": "8480-6",
              "display": "Systolic blood pressure"
            }
          ]
        },
        "valueQuantity": {
          "value": 120,
          "unit": "mmHg",
          "system": "http://unitsofmeasure.org",
          "code": "mm[Hg]"
        },
        "effectiveDateTime": "2024-03-14",
        "subject": {
          "display": "patient_test"
        }
      },
      "request": {
        "method": "POST",
        "url": "Observation"
      }
    },
    {
      "resource": {
```

```
        "resourceType": "Observation",
        "id": "jghjox6cu",
        "status": "final",
        "category": [
          {
            "coding": [
              {
                "system": "http://terminology.hl7.org/CodeSystem/observation-
category",
                "code": "vital-signs"
              }
            ]
          }
        ],
        "code": {
          "coding": [
            {
              "system": "http://loinc.org",
              "code": "8867-4",
              "display": "Heart rate"
            }
          ]
        },
        "valueQuantity": {
          "value": 103,
          "unit": "bpm",
          "system": "http://unitsofmeasure.org",
          "code": "/min"
        },
        "effectiveDateTime": "2024-11-07",
        "subject": {
          "display": "patient_test"
        }
      },
      "request": {
        "method": "POST",
        "url": "Observation"
      }
    }
  ]
}
```
